# Ante‐ and postmortem tau in autosomal dominant and late‐onset Alzheimer’s disease

**DOI:** 10.1002/acn3.51237

**Published:** 2020-11-05

**Authors:** Charles D. Chen, Timothy R. Holden, Brian A. Gordon, Erin E. Franklin, Yan Li, Dean W. Coble, Hongbo Luo, Randall J. Bateman, for the Dominantly Inherited Alzheimer Network (DIAN) and for the Dominantly Inherited Alzheimer Network Trials Unit (DIAN‐TU), Beau M. Ances, Richard J. Perrin, Tammie L. S. Benzinger, Nigel J. Cairns, John C. Morris

**Affiliations:** ^1^ Mallinckrodt Institute of Radiology Washington University in St. Louis St. Louis Missouri USA; ^2^ Department of Medicine Division of Geriatrics and Nutritional Science Washington University in St. Louis St. Louis Missouri USA; ^3^ Department of Pathology and Immunology Washington University in St. Louis St. Louis Missouri USA; ^4^ Department of Neurology Washington University in St. Louis St. Louis Missouri USA; ^5^ Division of Biostatistics Washington University in St. Louis St. Louis Missouri USA; ^6^ Department of Neurology Fifth Affiliated Hospital of Zunyi Medical University Zhuhai China; ^7^ College of Medicine and Health University of Exeter Exeter UK

## Abstract

Antemortem tau positron emission tomography imaging suggests elevated tau pathology in autosomal dominant versus late‐onset Alzheimer’s disease at equivalent clinical stages, but does not implicate the specific tau pathologies responsible. Here we made stereological measurements of tau neurofibrillary tangles, neuritic plaques, and neuropil threads and found compared to late‐onset Alzheimer’s disease, autosomal dominant Alzheimer’s disease showed even greater tangle and thread burdens. Regional tau burden resembled that observed in tau imaging of a separate cohort at earlier clinical stages. Finally, our results suggest tau imaging measures total tau burden in Alzheimer’s disease, composed predominantly of tangle and thread pathology.

## Introduction

Antemortem tau positron emission tomography (PET) imaging suggests elevated tau pathology in autosomal dominant (ADAD) versus late‐onset Alzheimer’s disease (LOAD) at equivalent clinical stages. Compared to LOAD, ADAD has shown elevated ^18^F‐flortaucipir^1^ radioligand binding in prefrontal, premotor, and inferior parietal cortices,^2^ as well as precuneus and lateral parietal cortices.^3^ However, PET imaging does not implicate specific tau pathologies responsible. Previous work quantitatively comparing AD tau pathology with PET imaging has typically been performed in a single individual,^4^, ^5^ and it is not known whether these results generalize, given the disease heterogeneity of both ADAD^6^ and LOAD^7^. To investigate which tau pathologies contribute to elevated ^18^F‐flortaucipir binding in ADAD versus LOAD cohorts, we made stereological measurements of three major features of AD tau pathology: neurofibrillary tangles, neuritic plaques, and neuropil threads.

## Methods

Protocols for the study have received prior approval by the local Institutional Review Board of each Dominantly Inherited Alzheimer Network site. Informed consent was obtained from each participant.

Cases selected for postmortem study were participants in the Dominantly Inherited Alzheimer Network (*n* = 7) or in studies directed by the Knight Alzheimer Disease Research Center (*n* = 10) (Table 1). These individuals met the inclusion criteria of high AD neuropathological change^8^ without comorbid neurodegenerative or vascular disease.

**Table 1:** Cohort demographics.

	Neuropathology cohort		Imaging cohort	
	LOAD	ADAD	LOAD	ADAD
Number	10	7	35	14
Age at visit, years (SD)			74.9 (6.75)	50 (12.5)
Age at onset, years (SD)	63.1 (9.83)	38.4 (4.65)		48.3 (0.83)^1^
Age at death, years (SD)	73.4 (8.29)	44.9 (7.47)		
Female (%)	6 (60%)	4 (57.1%)	19 (54.3%)	8 (57.1%)
MMSE at visit, score (SD)			25.3 (3.88)	21.9 (6.40)
CDR at visit, score (0/0.5/1/2/3)			0.657 (0/26/8/1/0)	0.714 (0/12/1/0/1)
CDR at death, score (0/0.5/1/2/3)	2.75 (0/0/1/0/7)	3 (0/0/0/0/6)		
*APOE* ε4 (%)	7/9 (77.8%)	1/7 (14.3%)	22/34 (64.7%)	4/14 (28.6%)
Family Mutation *APP*/*PSEN1*/*PSEN2*		0/7/0		1/12/1
Aβ plaque score (A0/1/2/3)	3 (0/0/0/10)	3 (0/0/0/7)		
NFT stage (B0/1/2/3)	3 (0/0/0/10)	3 (0/0/0/7)		
Neuritic plaque score (C0/1/2/3)	2.9 (0/0/1/9)	3 (0/0/0/7)		

^1^ Includes estimated age at onset using expected years to symptom onset (EYO).

Neuropathological assessment of cases involved expert evaluation of histology slides representing 16 brain areas from the left side of each brain.^9^ Stereology focused on tissues sampled in the coronal plane, including the frontal lobe (middle frontal gyrus), temporal lobe (superior and middle temporal gyri), parietal lobe (inferior parietal lobe including angular gyrus), occipital lobe (calcarine sulcus and peristriate cortex), parahippocampal gyrus, and hippocampal subfield CA1 (both sampled at the level of the lateral geniculate nucleus). From these regions, stereological measurements of PHF‐1 (a gift from Dr. Peter Davies) immunostained tangles, plaques, and threads were made using the Area Fraction Fractionator probe in Stereo Investigator 10 (MBF Bioscience, Williston, VT, USA).

In a separate cohort (ADAD *n* = 14, LOAD *n* = 35), antemortem ^18^F‐flortaucipir PET was quantified using regional standardized uptake value ratios (SUVRs).^10^ These individuals met the inclusion criteria of having a Clinical Dementia Rating (CDR®)^11^ greater than 0; individuals with LOAD additionally had positive amyloid PET imaging.^12^ Regional SUVRs of interest were defined by FreeSurfer^13^ regions best corresponding to neuropathology regions: caudal middle frontal cortex, middle temporal cortex, inferior parietal cortex, pericalcarine cortex, parahippocampal cortex, and hippocampus.

Regional differences across and within ADAD and LOAD in neuropathology and imaging were assessed using the Kruskal–Wallis test. Post hoc Wilcoxon rank‐sum tests were performed with Bonferroni–Holm multiple comparisons correction to assess which regions showed elevated tau burden in ADAD versus LOAD.

## Results

**Figure 1:**
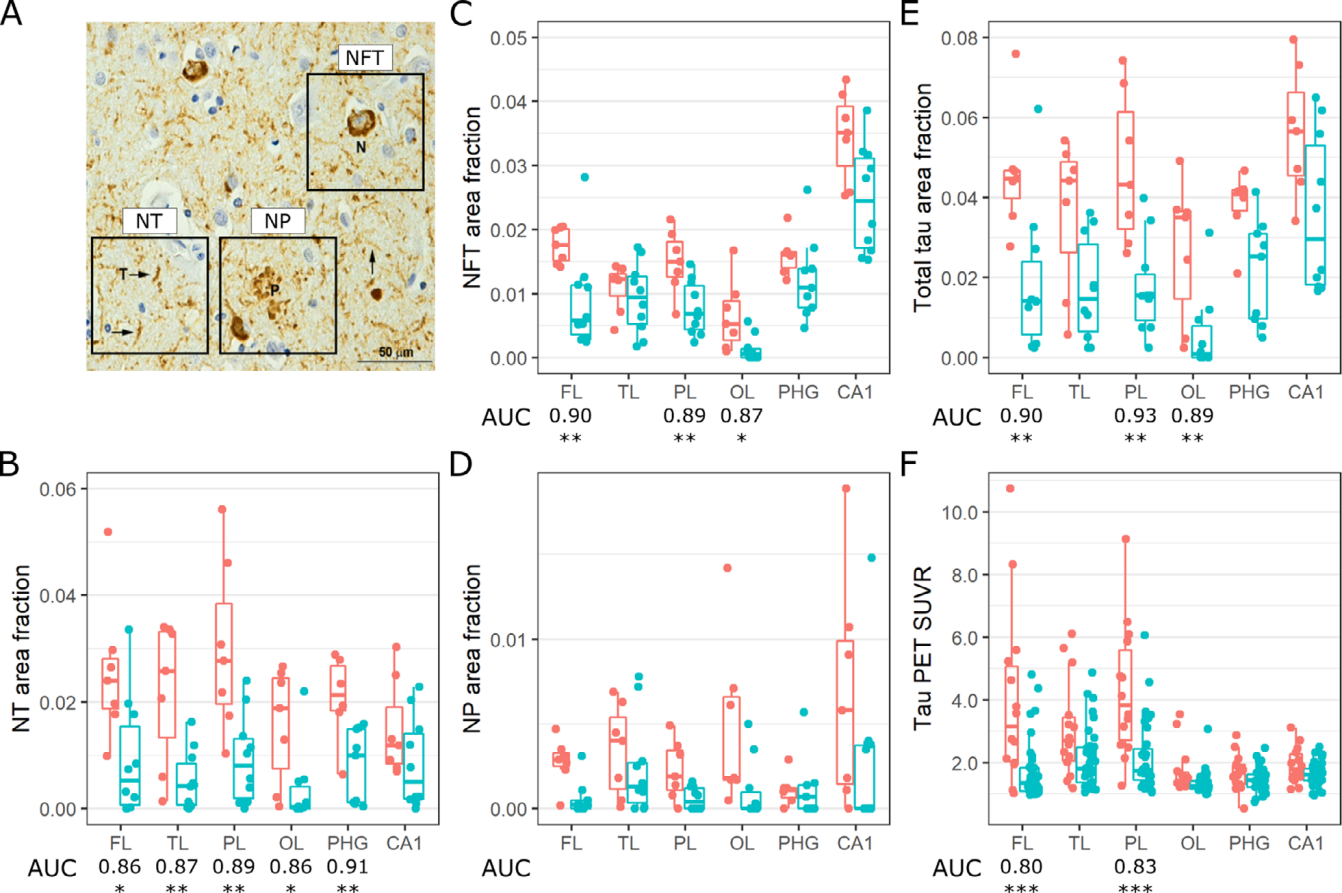
(A) Exemplar PHF‐1 immunostained neuropil threads (NT), neurofibrillary tangles (NFT), and neuritic plaques (NP). (B) Regional NT burden in the frontal lobe (FL), temporal lobe (TL), parietal lobe (PL), occipital lobe (OL), parahippocampal gyrus (PHG), and hippocampal subfield CA1 in ADAD and LOAD. (C) Regional NFT burden. (D) Regional NP burden. (E) Regional total tau (NT + NFT+NP) burden. (F) Regional ^18^F‐flortaucipir PET imaging SUVRs. Asterisks denote *P*‐values < 0.05 (*), 0.01 (**), and 0.001 (***) for regionwise Wilcoxon rank‐sum tests between ADAD and LOAD. Only area under the curves (AUCs, the probability that a randomly selected ADAD individual has a higher regional tau burden than a randomly selected LOAD individual) that are statistically significant after Bonferroni–Holm multiple comparisons correction are displayed.

Tangle, plaque, thread burden, and SUVR showed statistically significant regional differences across ADAD and LOAD. Only tangle burden and SUVR showed significant regional differences within ADAD and LOAD as well (Fig. 1).

Compared to LOAD, tangle burden in ADAD was significantly elevated outside the temporal lobe. Patterns of regional tangle burden resembled those of regional SUVRs. However, while CA1 is highest in median tangle burden, the hippocampus has only the third and fourth highest median SUVR in ADAD and LOAD, respectively (Fig. 1C&F).

Neuritic plaque burden was elevated in ADAD, but no post hoc test reached statistical significance after multiple comparisons correction. Thread burden was elevated outside CA1 (Fig. 1B&D).

## Discussion

Antemortem tau PET SUVRs and postmortem tangle burden in frontal and parietal regions were elevated in ADAD versus LOAD. This concordance appears robust at the group level, even though antemortem imaging and postmortem neuropathology were assessed in different cohorts, roughly representing early and late clinical stages of AD, respectively (Table 1). Additionally, the ADAD neuropathology cohort demonstrates an earlier age of onset than the ADAD imaging cohort, suggesting more aggressive forms of AD pathology in the former.

Given differences in clinical stage and age of onset, it is not surprising there are also discordant findings. First, in ADAD, tangle burden is elevated in the occipital lobe relative to LOAD, but SUVR is not. Second, medial temporal lobe regions show some of the highest regional tangle burden in the neuropathology, but not imaging, cohort. There are several potential explanations. First, tau burden may be particularly modest in the medial temporal lobe at early symptomatic stages of AD, but increase substantially by end stage. Second, PET imaging may have difficulty resolving the tau burden of small brain structures compared to histopathological assessment. Finally, some individuals in the imaging cohort may have subtle neuropathological comorbidities that contribute to cognitive impairment, qualifying an amyloid PET positive case with low AD neuropathological change (transentorhinal versus limbic stages of tau pathology) for inclusion in this study.

More discordances between imaging and neuropathology come from patterns of regional neuritic plaque and thread burden. Plaque burden was elevated in ADAD, though no post hoc test reached statistical significance, and thread burden was elevated outside CA1. Ringman *et al*.^14^ found statistically significant elevation of plaque burden in a larger cohort (ADAD *n* = 60, LOAD *n* = 120), but used a semi‐quantitative global score for each individual. We could not find any published studies comparing levels of neuropil threads between ADAD and LOAD. We also attribute these discordant findings to differences in clinical stage between neuropathology and imaging cohorts.

Comparing frontal, temporal, and parietal lobe values suggests tau PET SUVRs may correspond best to total tau burden (summed contributions from tangles, plaques, and threads). Similarly, Smith *et al*.^4^ found regional SUVRs correlated best with regional total tau burdens in a single individual with ADAD. However, that study also found threads outnumbered tangles in every studied brain region; we found no significant statistical dominance of thread over tangle burden in any region in either ADAD or LOAD.

Given our findings, we can make two conservative claims. First, although tau PET did not assess individuals in late stages of AD, and our neuropathologic assessments focused on very late stages, the regional pattern of elevated tau radioligand binding is largely concordant with the regional pattern of elevated postmortem total tau burden in ADAD versus LOAD. This suggests regional differences in tau pathology between ADAD and LOAD are consistent throughout their symptomatic stages. We propose that tau PET did not identify the relatively high tau burdens seen in the neuropathological assessment of CA1 and the parahippocampal gyrus because, at the level of the lateral geniculate nucleus, these areas develop far more robust tauopathy only in late stages of AD neuropathological change,^15^, ^16^ that are more likely to be associated with a terminal Clinical Dementia Rating of 3 than 0.5 or 1. In contrast, the entire hippocampus was assessed in tau PET imaging, which may explain some of the discordance in this comparison.

Second, like tangle burden, thread burden is elevated in ADAD versus LOAD, and across more brain regions, while plaque burden is elevated to a lesser extent. A possible explanation for greater tangle and thread burden in ADAD than LOAD might be that LOAD is often a multifactorial process, with cerebral small vessel disease, TDP‐43, and other co‐pathologies contributing to the clinicopathological phenotype such that less AD neuropathologic change is needed to reach similar states of dementia severity. That said, enhancement of tangle and thread burden in ADAD compared to LOAD without an equally strong enhancement of plaque burden may seem unusual. One explanation suggests tangles and threads are pathophysiologically closely linked, with tangles appearing first, and threads reflecting more severe saturation of neuronal processes by abnormal tau, whereas neuritic plaques develop later,^16^ and reflect more focal disturbances that leave remaining neuronal cytosol unperturbed. Another explanation: on sections immunostained for tau, within areas of very dense threads, plaques are occasionally difficult to discern, and might be undercounted.

We note the limitations to this study. First, no individuals in our neuropathology cohort had undergone antemortem tau PET, precluding direct imaging‐neuropathology comparisons. Second, regions included in the neuropathology portion of this study were limited in number and not perfectly correspondent to those from tau PET. Third, most ADAD individuals who came to autopsy were at the end stage of disease. Finally, there is a difference in age of AD symptom onset between the imaging and neuropathology cohorts. Earlier ages of onset appear to be correlated with higher cortical tau PET signal^17^, ^18^, ^19^ and thus there may be a mixed contribution of mutation and early age of onset to the tau PET imaging of the ADAD cohort. From the current study, the tau pathologies responsible for differences observed in tau PET between ADAD versus LOAD were revealed to be predominantly neurofibrillary tangles and neuropil threads. However, our current findings cannot address how temporal progression of tau pathology in ADAD differs from that in LOAD (hypothesized to begin in the brain stem^20^, ^21^ and suspected to share early‐stage distribution in the medial temporal lobe with primary age‐related tauopathy^22^). Future work can investigate the temporal progression of AD tau pathology more broadly by studying the relationship between earlier/later ages of AD symptom onset and tau pathology. One possibility is to introduce an early‐onset sporadic AD cohort to help disentangle the contributions of an earlier age of onset form the specific mutations that define the ADAD cohort.

## Author Contributions

B.A.G., E.E.F., H.L., R.J.B., B.M.A., R.J.P., T.L.S.B., N.J.C, and J.C.M. were involved in the conception and design of the study. T.R.H. was involved in the acquisition of the data. T.R.H., Y.L., and D.W.C. were involved in the preliminary analysis of the data. C.D.C. was involved in performing the final analysis of the data, writing the manuscript, and making the figures.

The composition of the DIAN study group is listed below.

**Table:** 

Carlos Cruchaga, PhD	Department of Psychiatry, Washington University in St. Louis, St. Louis, MO, USA
Anne Fagan, PhD	Department of Neurology, Washington University in St. Louis, St. Louis, MO, USA
Alison Goate, DPhil	Department of Genetics and Genomic Sciences, Ichan School of Medicine at Mount Sinai, New York, NY, USA
Jason Hassenstab, PhD	Department of Neurology, Washington University in St. Louis, St. Louis, MO, USA
Celeste Karch, PhD	Department of Psychiatry, Washington University in St. Louis, St. Louis, MO, USA
Eric McDade, DO	Department of Neurology, Washington University in St. Louis, St. Louis, MO, USA
Chengjie Xiong, PhD	Department of Neurology, Washington University in St. Louis, St. Louis, MO, USA

The composition of the DIAN‐TU study group is listed below.

**Table:** 

James J. Lah, MD, PhD	Department of Neurology, Emory University, Atlanta, GA, USA
Sarah B. Berman, MD, PhD	Department of Neurology, University of Pittsburgh, Pittsburgh, PA, USA
Jared R. Brosch, MD	Department of Neurology, Indiana University, Indianapolis, IN, USA
Ghulam Surti, MD	Department of Psychiatry and Human Behavior, Warren Alpert Medical School of Brown University, Providence, USA
Christopher H. van Dyck, MD	Alzheimer’s Disease Research Unit, Yale University School of Medicine, New Haven, CT, USA
Serge Gauthier, MD	McGill Center for Studies in Aging, Douglas Hospital, Montreal, Canada
Mario Masellis, MD, PhD	Division of Neurology, Department of Medicine, Sunnybrook Health Sciences Centre, Toronto, Canada

## Conflicts of Interest

R.J.B. is the principal investigator of the DIAN‐TU, which is supported in part by the DIAN‐TU Pharma Consortium. Eli Lilly and Company and Hoffman‐LaRoche, two members of the DIAN‐TU Pharma Consortium, provided funding for the DIAN‐TU‐001 trial, the former additionally providing technology transfer and precursor for ^18^F‐flortaucipir, the tau PET radioligand used in this study, and the latter additionally providing payment and reimbursement for speaking fees, advisory boards, and travel expenses of R.J.B. Additionally, B.A.G., Y.L., D.W.C., R.J.P., and T.L.S.B. are also members of the DIAN‐TU, and T.L.S.B. additionally participates as a site investigator in clinical trials sponsored by Eli Lilly and Company.
